# Supplementary material for: Breastfeeding Education and Support to Improve Early Initiation and Exclusive Breastfeeding Practices and Infant Growth: A Cluster Randomized Controlled Trial from a Rural Ethiopian Setting
Source: Nutrients. 2021 Apr 6;13(4):1204. doi: 10.3390/nu13041204 (PMC8067429; doi:10.3390/nu13041204)
Supplement: Supplementary file 1 [file nutrients-13-01204-s001.pdf]

## Supplementary Material

**Table S1:** Types of peer support intervention provided to women in the intervention arm

| Type of support | Activities |
|---|---|
| **Informational support** involves advice and suggestions | Encouraged delivery at the nearby health centre<br>Emphasized the importance of initiating breastfeeding within 1 hr of delivery and feeding colostrum first<br>Discouraged use of traditional pre-lacteal foods and post-lacteal foods<br>Encouraged the mother to increase their usual food intake to support lactation<br>Emphasised frequent and on-demand breastfeeding,<br>Encouraged the mothers to continue EBF for 6 months<br>Lactation amenorrhea method and other family planning options<br>Personal cleanliness and domestic hygiene, hand washing |
| **Emotional support** involves providing empathy, love, and care, built on relationships of trust | Discuss personal difficulties with breastfeeding<br>Showed expressions of caring, encouragement, attentive listening, reflection, reassurance, and avoiding critics.<br>This support would foster the experience of feeling accepted, cared for, admired, empathized, respected, and valued. |
| **Instrumental support** consists of providing tangible aid | Observed the positioning, the latching, and the feeding of the new-born with hands-on guidance as necessary<br>Solved any breastfeeding problems<br>Helped breast milk expression and storage |
| **Appraisal support** facilitates self-evaluation through constructive feedback | Provided constructive/ appropriate feedback<br>Praised the mother for correct actions<br>Encouraged to persist in problem-solving<br>Reassured that their efforts will result in positive outcomes |

**Table S2:** Maternal knowledge, attitude and practices related to breastfeeding by study arms at 1 month or 6 months postpartum follow-ups[1]

| Outcomes | Intervention (*n* = 212) | Control (*n* = 197) | Unadj diff (95% CI)[2] | *P*[2] | Adj diff (95% CI)[2] | |
|---|---|---|---|---|---|---|
| Early initiation of breastfeeding[3] | 68.9 | 43.1 | 25.9 (13.8, 37.9) | <0.001 | 25.6 (13.7, 37.4) | <0.001 |
| Exclusive breastfeeding[4] | 64.6 | 50.3 | 14.4 (3.93, 24.8) | 0.008 | 14.2 (2.73, 25.7) | 0.017 |
| BFKQ score[4] | 26.4 ± 1.91 | 26.0 ± 2.19 | 0.16 (-0.09, 0.40) | 0.211 | 0.16 (-0.09, 0.41) | 0.222 |
| IIFAS score[4] | 78.5 ± 9.14 | 67.3 ± 9.89 | 0.87 (0.73, 1.00) | <0.001 | 0.87 (0.72, 1.01) | <0.001 |
| Good knowledge about breastfeeding[4] | 71.7 | 66.5 | 5.20(-38.0, 14.2) | 0.257 | 5.38 (-35.9, 14.4) | 0.239 |
| Positive attitude towards breastfeeding[4] | 83.0 | 44.7 | 38.3 (29.7, 46.9) | <0. 001 | 38.2 (29.5, 46.8) | <0.001 |

[1]Values are mean ± SD or proportions

[2]Unadjusted and adjusted group difference and P's estimated using linear regression models (as mean difference) for the continuous outcomes and linear probability models (as risk difference in percentage points) for proportions, with a robust variance estimation used to account for clustering of subjects by sub-districts. Covariates used for adjusted estimates were maternal age, educational status, wealth index, parity, and IIFAS at baseline.

[3]Assessed at 1 month postpartum

[4]Assessed at 6 months postpartum

BFKQ, Breast Feeding Knowledge Questionnaire; IIFAS, Iowa Infant Feeding Attitude Scale

**Table S3:** Infant anthropometry and morbidity outcomes by study arms at 6 months postpartum follow-up[1]

| Variable | Intervention (*n = 212)* | Control (*n = 197)* | Unadj diff (95% CI)[2] | *P*[2] | Adj diff (95% CI)[2] | *P*[2] |
|---|---|---|---|---|---|---|
| LAZ | -0.13 ± 1.25 | -0.18 ± 1.29 | 0.05 (-0.30, 0.39) | 0.795 | 0.05 (-0.30, 0.39) | 0.790 |
| WAZ | 0.03 ± 1.02 | -0.12 ± 1.11 | 0.15 (-0.12, 0.41) | 0.267 | 0.15 (-0.11, 0.41) | 0.255 |
| WLZ | 0.25 ± 1.21 | 0.09 ± 1.13 | 0.15 (-0.16, 0.46) | 0.326 | 0.15 (-0.15, 0.46) | 0.311 |
| MUAC | 13.7 ± 1.04 | 13.5 ± 0.98 | 0.25 (0.00, 0.50) | 0.048 | 0.25 (0.01, 0.49) | 0.040 |
| Stunted | 5.66 | 7.11 | -1.45 (-6.90, 4.01) | 0.594 | -1.51 (-6.84, 3.81) | 0.568 |
| Underweight | 3.77 | 5.08 | -0.31 (-1.29, 0.68) | 0.537 | -0.28 (-1.38, 0.82) | 0.618 |
| Wasted | 4.72 | 1.52 | 1.16 (0.03, 2.29) | 0.044 | 1.16 (0.00, 2.33) | 0.051 |
| Cough | 19.8 | 26.9 | -7.09 (-15.2, 1.04) | 0.085 | -7.29 (-15.3, 0.66) | 0.071 |
| Fever | 18.9 | 23.9 | -4.98 (-12.6, 2.58) | 0.189 | -4.79 (-12.2, 2.64) | 0.199 |
| Diarrhea | 11.8 | 9.14 | 2.66 (-4.00, 9.31) | 0.424 | 2.73 (-3.99, 9.44) | 0.416 |
| Fever with cough | 13.9 | 20.6 | -6.72 (-13.4, -0.05) | 0.048 | -6.96 (-13.3, -0.62) | 0.032 |
| Any illness | 32.6 | 38.6 | -6.03 (-16.3, 4.23) | 0.241 | -6.08 (-16.1, 3.94) | 0.226 |
| Serious illness | 22.2 | 30.5 | -8.29 (-17.5, 0.96) | 0.077 | -7.91 (-17.0, 1.19) | 0.087 |

[1]Values are mean ± SD or proportions

[2]Unadjusted and adjusted group differences (CI's) and *P*'s estimated using linear regression models (as means difference) for the continuous outcomes, logistic regression models (as odds ratio) for the nutritional status outcomes, and linear probability models (as risk difference in percentage points) for the morbidity outcomes, with a robust variance estimation used to account for clustering of subjects by sub-districts. Covariates used for adjusted estimates were maternal age, educational status, wealth index, parity, IIFAS, and household food insecurity status at baseline

LAZ, length-for-age *z* scores; MUAC, mid-upper-arm-circumference in cm; WAZ, weight-for-age *z* scores; WHZ, weight-for-length *z* scores
